# Dicyandiamide-Driven Tailoring of the *n*-Value Distribution and Interface Dynamics for High-Performance ACI 2D Perovskite Solar Cells

**DOI:** 10.1007/s40820-025-01817-x

**Published:** 2025-06-23

**Authors:** Ge Chen, Yunlong Gan, Shiheng Wang, Xueru Liu, Jing Yang, Sihui Peng, Yingjie Zhao, Pengwei Li, Asliddin Komilov, Yanlin Song, Yiqiang Zhang

**Affiliations:** 1 College of Chemistry, Zhengzhou University, Zhengzhou, 450001 People’s Republic of China; 2 Karshi State Technical University, 18100 Karshi, Uzbekistan; 3 National Scientific Research Institute of Renewable Energy Sources, 100084 Tashkent, Uzbekistan; 4 Key Laboratory of Green Printing, CAS Research/Education Center for Excellence in Molecular Sciences, Institute of Chemistry, Beijing Engineering Research Center of Nanomaterials for Green Printing Technology, National Laboratory for Molecular Sciences (BNLMS), Chinese Academy of Sciences (ICCAS), Beijing, 100190 People’s Republic of China

**Keywords:** Alternating-cation-interlayer 2D perovskite solar cell, Phase modulation, Buried interface, Interface dynamics

## Abstract

Organic–inorganic hybrid perovskite solar cells achieve remarkable efficiencies (> 26%) yet face stability challenges. Quasi-2D alternating-cation-interlayer perovskites offer enhanced stability through hydrophobic spacer cations but suffer from vertical phase segregation and buried interface defects. Herein, we introduce dicyanodiamide (DCD) to simultaneously address these dual limitations in GA(MA)_n_Pb_n_I_3n+1_ perovskites. The guanidine group in DCD passivates undercoordinated Pb^2+^ and MA^+^ vacancies at the perovskite/TiO_2_ interface, while cyano groups eliminate oxygen vacancies in TiO_2_ via Ti^4+^–CN coordination, reducing interfacial trap density by 73% with respect to the control sample. In addition, DCD regulates crystallization kinetics, suppressing low-n-phase aggregation and promoting vertical alignment of high-n phases, which benefit for carrier transport. This dual-functional modification enhances charge transport and stabilizes energy-level alignment. The optimized devices achieve a record power conversion efficiency of 21.54% (vs. 19.05% control) and retain 94% initial efficiency after 1200 h, outperforming unmodified counterparts (84% retention). Combining defect passivation with phase homogenization, this work establishes a molecular bridge strategy to decouple stability-efficiency trade-offs in low-dimensional perovskites, providing a universal framework for interface engineering in high-performance optoelectronics.

**Supplementary Information:**

The online version contains supplementary material available at 10.1007/s40820-025-01817-x.

## Highlights

- Guanidine derivatives of dicyanodiamide (DCD) is introduced into the buried interface of quasi-2D alternating-cation-interlayer perovskites (GA(MA)_n_Pb_n_I_3n+1_), which simultaneously realize the defect passivation of buried interface and the regulation of phase distribution.
- This interface engineering not only reduces interfacial defects but also enhances the interfacial charge transfer, and the DCD-regulated perovskite solar cells exhibit a significant increase in efficiency from 19.05% to 21.54%.

**Supplementary Information:**

The online version contains supplementary material available at 10.1007/s40820-025-01817-x.

## Graphical abstract

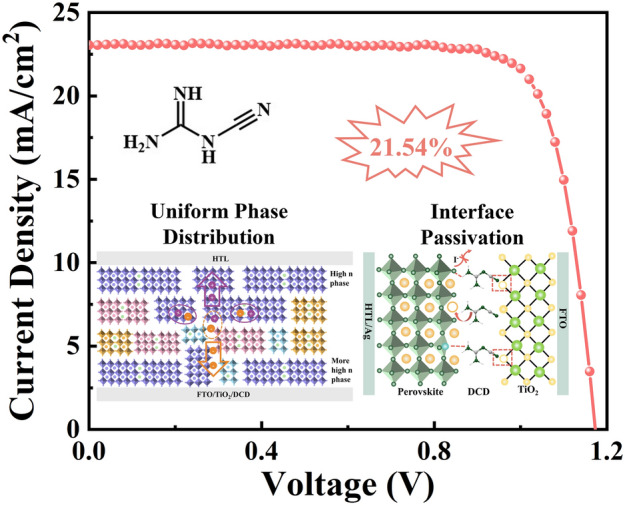

## Introduction

Organic–inorganic hybrid perovskite solar cells (PSCs) have emerged as a transformative photovoltaic technology due to their exceptional optoelectronic properties—including tunable bandgaps, high absorption coefficients (> 10^5^ cm^−1^), and ambipolar charge transport—combined with cost-effective solution processability [1, 2]. While inverted 3D PSCs have achieved a certified power conversion efficiency (PCE) of 26.7% through advances in crystallization control and defect passivation, their commercial viability remains constrained by the inherent instability of perovskite materials under operational stressors (thermal cycling, humidity, and ion migration) [3–6].

To address this limitation, structural engineering of perovskites using hydrophobic spacer cations (e.g., BA^+^, PEA^+^) has enabled the development of 2D layered halide perovskites with enhanced moisture resistance and suppressed ion migration through two mechanisms: (i) steric hindrance from organic spacers reduces water permeation pathways, and (ii) strong hydrogen bonding between spacer cations and inorganic slabs inhibits halide migration [7–12]. These materials crystallize into three primary architectures: Ruddlesden-Popper (RP, monovalent spacers), Dion-Jacobson (DJ, divalent spacers), and alternating cation interlayer (ACI, dual-cation spacers) [13, 14]. The ACI-type structure distinguishes itself through its unique [PbI_6_]^4−^ octahedral connectivity—where adjacent inorganic layers are bridged by two distinct organic cations (e.g., GA^+^ and MA^+^) alternating in the interlayer space [15]. This configuration, first explored by Kanatzidis et al. using guanidinium ions (GA^+^), reduces the van der Waals gap to < 3 Å (vs. ~ 5 Å in RP-type), enabling efficient carrier transport through enhanced interlayer *π*-orbital overlap and phonon-assisted tunneling [16]. Density functional theory (DFT) calculations further reveal that ACI perovskites exhibit a 30% lower exciton binding energy (E_b_ ≈ 120 meV) compared to RP counterparts, combined with a near-ideal bandgap of 1.65 eV for single-junction devices [17]. These inherent advantages have propelled ACI perovskites to record efficiencies of 22.26% in quasi-2D configurations via interfacial modifications, such as imidazolium iodide surface treatment [18].

Quasi-2D perovskites, characterized by mixed-phase nanostructures with vertically graded dimensionality (*n* values), offer an optimal compromise between stability and efficiency through quantum confinement effects [19–21]. However, solution-processed films typically exhibit heterogeneous phase distributions due to kinetically driven growth disparities: low-n domains (*n* = 1–3) nucleate preferentially at the air interface due to rapid solvent evaporation, while high-n phases (*n* ≥ 5) dominate near the substrate where crystallization is diffusion-limited [22]. Such vertical phase segregation creates substantial charge transport bottlenecks, as evidenced by conductive atomic force microscopy (c-AFM) showing 2–3 orders of magnitude lower conductivity in low-n regions [23]. In RP and DJ systems, strategies like vacuum poling (applying − 0.1 MPa during annealing to induce phase redistribution) [24] and thiourea additives (modulating crystallization kinetics via S·Pb coordination) [25] have improved homogeneity. Nevertheless, phase uniformity engineering in ACI-type perovskites remains underexplored, particularly regarding the dynamic interplay between spacer cation geometry and phase evolution during spin-coating.

A critical yet frequently neglected challenge lies in optimizing the buried perovskite/transport layer interface, which governs both interfacial defect density and perovskite crystallization dynamics [26–28]. Time-resolved photoluminescence (TRPL) studies reveal that non-radiative recombination at this interface accounts for > 60% of total carrier losses in quasi-2D devices. The origin of these losses can be traced to two defect types: (i) undercoordinated Pb^2+^ (acting as deep traps with activation energy ~ 0.8 eV) and (ii) iodide vacancies (V_I_, creating Urbach tail states) [29–31]. These defects reduce the quasi-Fermi level splitting (ΔEF) by 150–200 meV, directly limiting *V*_OC_ to < 1.1 V despite theoretical predictions of 1.3 V [32]. Furthermore, poor interfacial contact with the TiO_2_ electron transport layer (ETL) exacerbates series resistance and induces nonuniform perovskite crystallization [33]. Addressing these buried interfacial defects represents a crucial frontier for advancing quasi-2D PSCs.

In this work, we introduce guanidine derivatives of dicyanodiamide (DCD) into the buried interface of quasi-2D ACI perovskites (GA(MA)_n_Pb_n_I_3n+1_), which simultaneously realize the defect passivation of buried interface and the regulation of phase distribution. We observe that DCD treatment regulates the multi-phase distribution in 2D ACI perovskite, increasing the proportion of the high *n* value phase at the bottom of the film, thereby accelerating charge transport. And the guanidine group in DCD interacts with uncoordinated Pb^2+^ and I atoms, effectively passivating iodide vacancy defects and cation vacancies at the perovskite buried interface. Additionally, the cyano (–CN) group in DCD interacts with Ti^4+^ in the TiO_2_ electron transport layer, reducing oxygen vacancy defects and strengthening the contact between TiO_2_ and perovskite layers. Our results demonstrate that the DCD modification reduces defects in the buried interface of the perovskite and yields a more uniform phase distribution. As a result, the DCD-regulated PSCs exhibit a significant increase in efficiency from 19.05 to 21.54%, coupled with improved long-term stability.

## Experimental Section

### Materials

The substrates used in this study were patterned F-Doped tin oxide (FTO) with a sheet resistance of 15 Ω^−1^, which was provided by South China Science & Technology Co. High-purity reagents, including lead iodide (PbI_2_, 99.99%), methylamine iodide (MAI, 99.5%) were sourced from Advanced Election Technology CO., Ltd. Methylamine hydrochloride (MACl, 99.5%), 2,2′,7,7′-tetrakis(N,N-dip-methoxyphenylamine)-9,9′-spirobifluorene (Spiro-OMeTAD, 99.8%), 4-t-butylphenylammonium iodide (tBP), and lithium bis(trifluoromethanesulfonyl)imide (Li-TFSI, 99%) were purchased from Xi’an Polymer Light Technology Corp. Guanidinium iodide (GAI, 99%) was purchased from Sigma-Aldrich. Solvents used throughout the process, including N, N-dimethylformamide (DMF, 99.8%), dimethyl sulfoxide (DMSO, 99.7%) and chlorobenzene (CB, 99.8%), were sourced from Sigma-Aldrich. We obtained ethyl acetate (EA, 99.8%), titanium tetrachloride (TiCl_4_, 99.9%), Dicyanodiamide (DCD, 99%) from Aladdin.

### Solar Cell Fabrication

The FTO substrates were cleaned using deionized water, acetone, and ethanol in sequence, each step lasting 15 min. Afterward, the FTO glass substrates were blown with dry nitrogen gas and treated with ultraviolet ozone for 20 min. The TiO_2_ layer was fabricated by chemical bath deposition, where the cleaned FTO substrate was submerged in a mixed solution of TiCl_4_ and H_2_O with the volume ratio of TiCl_4_ to H_2_O = 0.0225:1 at 70 °C for 1 h and then heat-treatment at 100 °C for 1 h. The TiO_2_ substrate was exposed to ultraviolet ozone for 15 min before spin-coating the next layer. For fabricating DCD modified substrates, DCD solution was prepared by dissolving 2 mg DCD power in 1 mL DMF. Subsequently, 50 μL DCD solution was dropped on the surface of TiO_2_ then spin-coating at 4500 r min^−1^ for 30 s and then annealed at 100 °C for 5 min.

The 1.2 M precursor solution for GA(MA)_n_Pb_n_I_3n+1_ (*n* = 5) was prepared by mixing GAI, MAI, and PbI_2_with the molar ration of 1:5:5 in DMF and DMSO (10/1, v/v) under N_2_ condition. MACl was added as an additive into perovskite precursor solution with the concentration of 10 mg mL^−1^. The perovskite film was prepared by spin-coating at a low speed of 500 r min^−1^ for 3 s and a high speed of 4000 r min^−1^ for 60 s, where 300 μL of ethyl acetate was dropped onto the substrate at 45 s before the end of the spin-coating. The films were then annealed at 150 °C for 20 min. The Spiro-OMeTAD solution was prepared by dissolving 90 mg of Spiro-OMeTAD, 22 µL of a Li-TFSI solution (520 mg Li-TFSI in 1 mL acetonitrile), and 36 µL of tBP in 1 mL of chlorobenzene. This solution was then spin-coated onto the perovskite film at 4000 r min^−1^ for 30 s. Finally, a 60 nm thick layer of silver (Ag) was thermally evaporated as an electrode using a shadow mask to complete the device structure. The area of the small-sized device is 0.04 cm^2^.

### Characterization

The current density–voltage (*J*-*V*) tests were conducted using a B2901A source meter equipped with a solar simulator (Enlitech, SS-X50) under simulated sun illumination (AM 1.5G, 1 sun). The X-ray diffraction (XRD) was recorded on the Bruker X-ray diffractometer using Cu Kα. Scanning electron microscopy (SEM) images were captured using the Regulus 8100. UV–vis absorption spectra were measured by a Cary 5000 spectrophotometer (Agilent Technologies). The photoluminescence (PL) spectra were conducted by a fluorescence spectrometer (HORIBA FluoroMax). The ultrafast transient absorption (TA) spectroscopy was measured using the Ultrafast System femtosecond TA spectrometer, which is based on a Yb: KGW laser system. The X-ray photoelectron spectroscopy (XPS) data were obtained by an X-ray photoelectron spectrometer (Thermo Scientific K-Alpha). The external quantum efficiency (EQE) of devices was measured by QE-R of Enlitech. The transient photocurrent (TPC) and transient photovoltage (TPV) was measured by PAIOS of Fluxim.

## Results and Discussion

### Interaction Between DCD and TiO_2_, Perovskite Layer

**Fig. 1:**
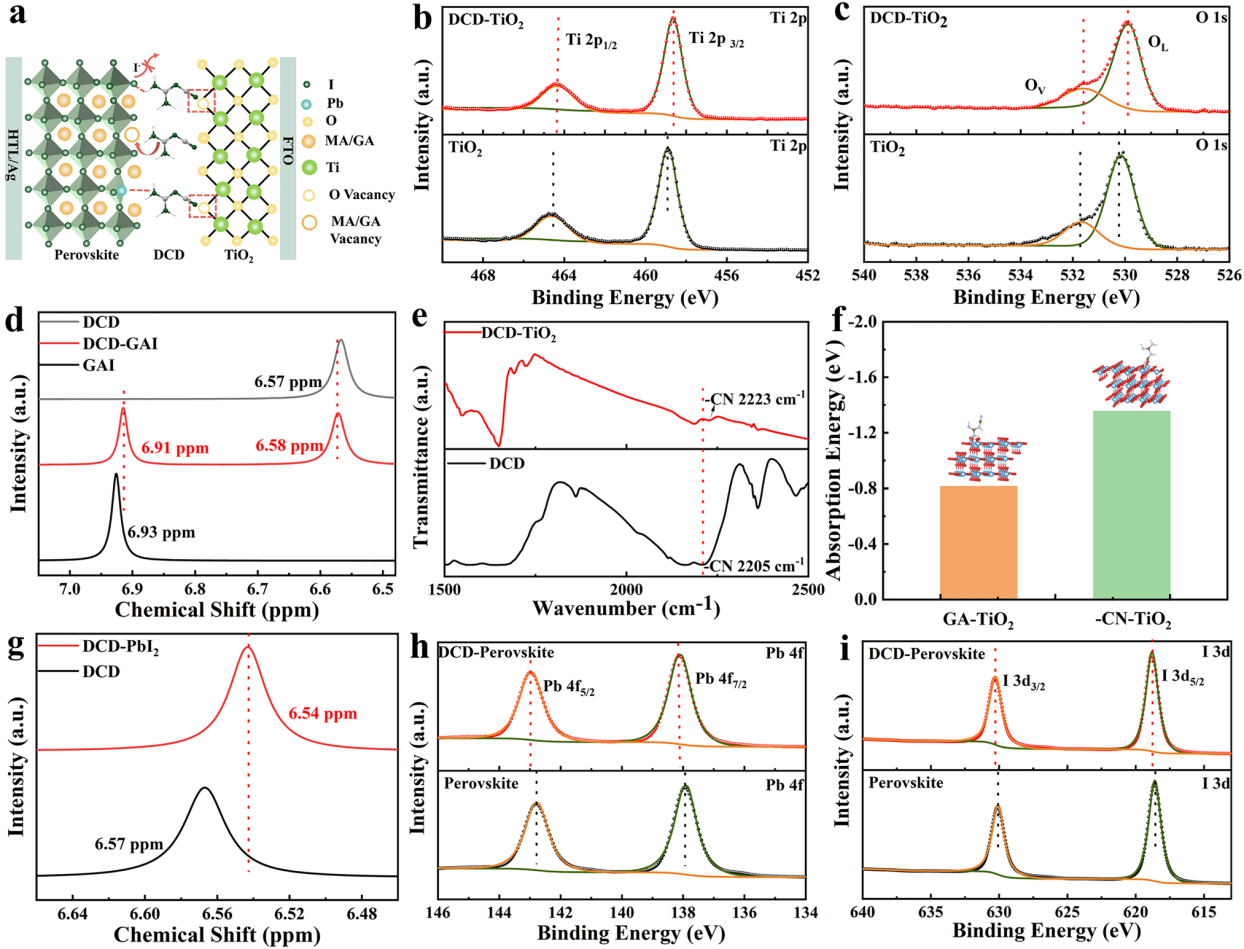
**a** Schematic illustration of the interfacial layer mechanism of DCD. XPS spectra of **b** Ti 2*p*, **c** O 1*s* of the TiO_2_ and DCD modified TiO_2_ films. **d** ^1^H NMR spectra of GAI, DCD and mixed powder of DCD and GAI. **e** FTIR spectra of pure DCD and DCD-TiO_2_. **f** Theoretical calculation of -CN and GA absorbed on TiO_2_ surface. **g** ^1^H NMR spectra of PbI_2_ and mixed powder of DCD and PbI_2_. XPS spectra of **h** Pb 4*f* and **i** I 3*d* of the exposed buried perovskite interface

Under continuous UV irradiation, the Ti^4+^-oxygen vacancy (V_O_) defect in the TiO_2_ layer can capture photoelectrons, which diminishes the device performance and accelerates the degradation of the perovskite structure [34]. In addition to these surface defects, the buried interface of perovskite also contains uncoordinated Pb^2+^ ions, cation vacancies, and antisite defects. To address these issues, we introduced dicyanodiamide (DCD) as an interfacial layer between the electron transport layer (ETL) and the perovskite layer (see Fig. S1 for the chemical structure). The cyano group (–CN) in DCD, containing lone-pair electrons, can coordinate with metal ions possessing vacant orbitals [35]. Moreover, the guanidine (GA) group in DCD is capable of interacting with uncoordinated Pb^2+^ ions and cation vacancies through electrostatic interactions and Lewis acid–base reactions. The GA group can also form hydrogen bonds with iodine atoms at the buried interface [36]. Thus, we hypothesize that the DCD molecules act as a bridge between the ETL and perovskite layers, enhancing the contact at the interface, facilitating the charge transport, and reducing defect states at the buried perovskite interface (Fig. 1a). We screened the concentration of the DCD and found that the concentration of 2 mg/mL was the best.

To validate this hypothesis, we employed XPS and Fourier transform infrared spectroscopy (FTIR) to investigate the presence and interaction of DCD with TiO_2_. The high-resolution XPS spectrum of the nitrogen element in DCD-TiO_2_ (Fig. S2) showed distinct new peaks, confirming the successful incorporation of DCD onto TiO_2_. Simultaneously, the characteristic –CN peak at 2223 cm^−1^ was clearly visible in the FTIR spectrum of the DCD-TiO_2_ mixture, further confirming the presence of DCD (Fig. S3). The Ti 2*p* XPS spectrum (Fig. 1b) revealed that the addition of DCD shifted the Ti 2*p*_3/2_ and Ti 2*p*_1/2_ peaks from 458.87 and 464.58 eV to 458.62 and 464.30 eV, respectively, indicating an interaction between DCD and Ti. This shift suggests that the DCD interaction enhances the electron density on Ti^4+^, further confirming the modification of TiO_2_ [37]. The O 1 s XPS spectra (Fig. 1c) show that the characteristic peaks for lattice oxygen (O_L_) at 530.10 eV and oxygen vacancy (O_V_) at 531.11 eV shifted to lower binding energies upon DCD modification. Additionally, the O_L_-to-O_V_ ratio changed from 52:48 to 67:33, indicating a reduction in oxygen vacancy defects within TiO_2_ upon DCD modification [38]. This suggest that the interaction between the DCD and Ti^4+^ plays a key role in reducing oxygen vacancies.

To further investigate the interaction between DCD and TiO_2_, DFT calculations were performed to compare the absorption energy of O_V_ on TiO_2_ with two groups from DCD (–CN and GA). As shown in Fig. 1f, the –CN group exhibited a lower adsorption energy (− 1.35 eV) than the GA group (− 0.81 eV) when interacting with oxygen vacancies on TiO_2_, supporting the preference of –CN for TiO_2_ interaction. This DFT results shows that –CN in DCD preferentially interacts with TiO_2_ through –CN–Ti bonding. FTIR analysis (Fig. 1e) further supports this, with the –CN stretching peak shifting from 2205 to 2223 cm^−1^ after DCD modification, suggesting the formation of a –CN–Ti interaction [39].

To explore the interaction between DCD and the perovskite layer, high-resolution XPS and ^1^H-nuclear magnetic resonance (NMR) spectroscopy were conducted. Figure 1d shows the NMR peaks for DCD at 6.57 ppm and GAI at 6.93 ppm. In the NMR spectrum shown in Fig. S4, the peaks corresponding to the hydrogen atoms in GA^+^ and DCD are labeled with red asterisks (*) and black hash symbols (#), respectively. In a mixed solution of DCD and GAI, the peaks shifted from 6.93 to 6.91 ppm for GAI and from 6.57 to 6.58 ppm for DCD, indicating that DCD acts as an electron donor, while GAI functions as an electron acceptor. The *π*–*π* interaction between DCD and GAI alters the chemical environment of the protons, resulting in a change in the H chemical shift [40]. This intermolecular *π*-*π* interaction between DCD and GAI is beneficial for charge-carrier transport in quasi-2D PSCs [41]. Additionally, when PbI_2_ was added to the DCD solution, the H signal of NMR spectrum for DCD shifted from 6.57 to 6.54 ppm (Fig. 1g), indicating an electrostatic interaction between the GA group of DCD and PbI_2_, affecting the chemical environment of the hydrogen atom [42]. XPS analysis of Pb and I elements at the buried interface of the perovskite layer (Fig. 1h) showed the binding energy of 4*f*_5/2_ and 4*f*_7/2_ for Pb^2+^ shifted from 142.76 and 137.90 eV to 142.95 and 138.05 eV, respectively, due to the electrostatic interaction between the DCD and [PbI_6_]^4−^. Similarly, the I 3*d*_3/2_ and I 3*d*_5/2_ peaks, initially positioned at 630.08 and 618.57 eV, respectively, shifted to 630.25 and 618.72 eV upon the introduction of DCD (Fig. 1i). These shifts suggested that the introduction of DCD alters the chemical environment of [PbI_6_]^4−^. Moreover, DCD reduced the formation of iodine vacancy defects, as shown by the calculation of the formation energy of I vacancy defects on the perovskite surface (Fig. S5). Therefore, we systematically prove that the GA group in DCD interacts with I to reduce the generation of I vacancies, and with [PbI_6_]^4−^ to decrease the formation of uncoordinated Pb defects and MA/GA cation vacancy defects at the buried interface of the perovskite [43, 44].

### Interface Optimization of TiO_2_ and Perovskite Layer

**Fig. 2:**
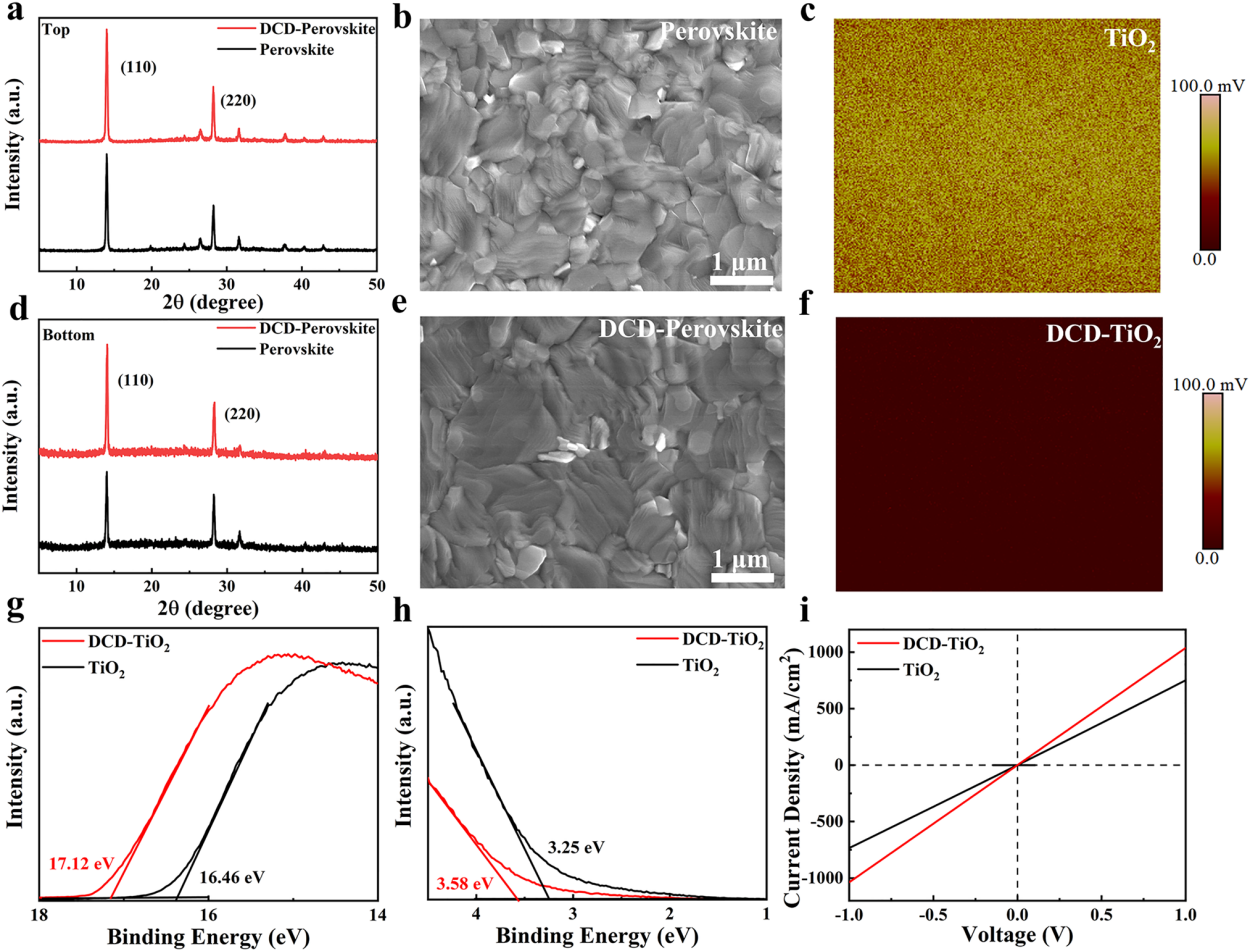
XRD patterns of **a** top surface, **d** bottom surface of perovskite films with and without modification. **b** SEM image of the top surface of perovskite films without DCD modified. **c** KPFM image of TiO_2_. **e** SEM image of the top surface of perovskite films with DCD modified. **f** KPFM image of DCD-TiO_2_. **g**, **h** UPS spectra of secondary electron cut-off and valence bands of TiO_2_ and DCD-TiO_2_ ETL. **i** Dark J-V cures of devices structured as FTO/ETLs/Ag

Next, we examined the influence of DCD on the morphology and electronic properties of TiO_2_ and perovskite films. Scanning electron microscopy (SEM) images of the top surfaces of perovskite films deposited on different substrates are shown in Fig. 2b, e, revealing that the DCD-modified perovskite film had a flatter top surface with larger grains compared to the unmodified film. Additionally, SEM images of the bottom surface (Fig. S6a, b), obtained through a delamination process, showed fewer voids in the DCD-modified perovskite film. The white spots in the images represent the residual TiO_2_ left during the peeling process. To quantify the roughness of the buried interface of perovskite, 3D optical profilometry (Fig. S7) was employed to confirm that the surface roughness of DCD-modified perovskite films was significantly reduced from 13.85 to 8.29 nm. This result corroborates the SEM observations of the bottom surface. The DCD treatment not only improved the morphology of perovskites but also significantly enhanced its crystallization. X-ray diffraction (XRD) results of the top and bottom perovskite surfaces showed a noticeable increase in the intensity of the (110) peak, indicating enhanced crystalline quality (Fig. 2a, d). Additionally, we performed ultraviolet-vis (UV–vis) absorption measurements on both the pristine perovskite film and the DCD-modified perovskite film. As shown in Fig. S8, the DCD-modified perovskite film exhibits a slight enhancement in absorbance, indicating an improvement in crystallization.

DCD not only optimized the crystalline quality of perovskite films but also improved the surface and electronic properties of TiO_2_. Atomic force microscopy (AFM) analysis (Fig. S9) of the TiO_2_ surface revealed that the DCD-modified TiO_2_ films had a reduced root mean square roughness (RMS) of 23.0 nm compared to 27.7 nm for the pure TiO_2_ films. The conductivity of TiO_2_ films with and without DCD was measured using a device structure of FTO/ETLs/Ag. The direct conductivity (*σ*) was calculated from the current–voltage curve (Fig. 2i), using the following formula [45]:

1 $$\sigma = \frac{Id}{{VA}}$$

where A is the area of the devices and d is the thickness of the films. The calculated conductivity of TiO_2_ and DCD-TiO_2_ was 5.94 × 10^–3^ and 8.19 × 10^–3^ mS cm^−1^, respectively. This enhanced conductivity of TiO_2_-DCD films improves the charge extraction and transport efficiency in perovskite solar cells. The Kelvin probe force microscopy (KPFM) was used to characterize the surface potential of the films (Fig. 2c, f). The TiO_2_-DCD film exhibited a darker color, indicating an increased Fermi level compared to pure TiO_2_, further supporting the improved compatibility between the ETL and perovskite layers. Ultraviolet photoelectron spectroscopy (UPS) was employed to characterize the accurate work function of the films. After depositing DCD on TiO_2_, the secondary electron cut-off edge shifted from 16.46 to 17.12 eV, and the corresponding work function value shifted from − 4.76 to − 4.1 eV (Fig. 2g), consistent with the KPFM results. From Fig. 2h, we obtained the valence band maximum energy (E_VBM_) of TiO_2_ (− 8.01 eV) and DCD-TiO_2_ (− 7.68 eV). Combining this data with the Tauc plots of TiO_2_ and TiO_2_-DCD (Fig. S10), the conduction band minimum energy (E_CBM_) of TiO_2_ and DCD-TiO_2_ was found to be − 4.17 and − 3.88 eV, respectively. Comparing these energy level values, we drew the energy level alignment diagram (Fig. 4a) of perovskite solar cells [18]. The diagram shows that TiO_2_ regulated by DCD is better aligned with the perovskite layer, facilitating improved charge extraction and transport between the electron transport layer (ETL) and the perovskite layer. This explains the enhanced performance of DCD-regulated PSCs.

### Phase Distribution of Perovskite Film

**Fig. 3:**
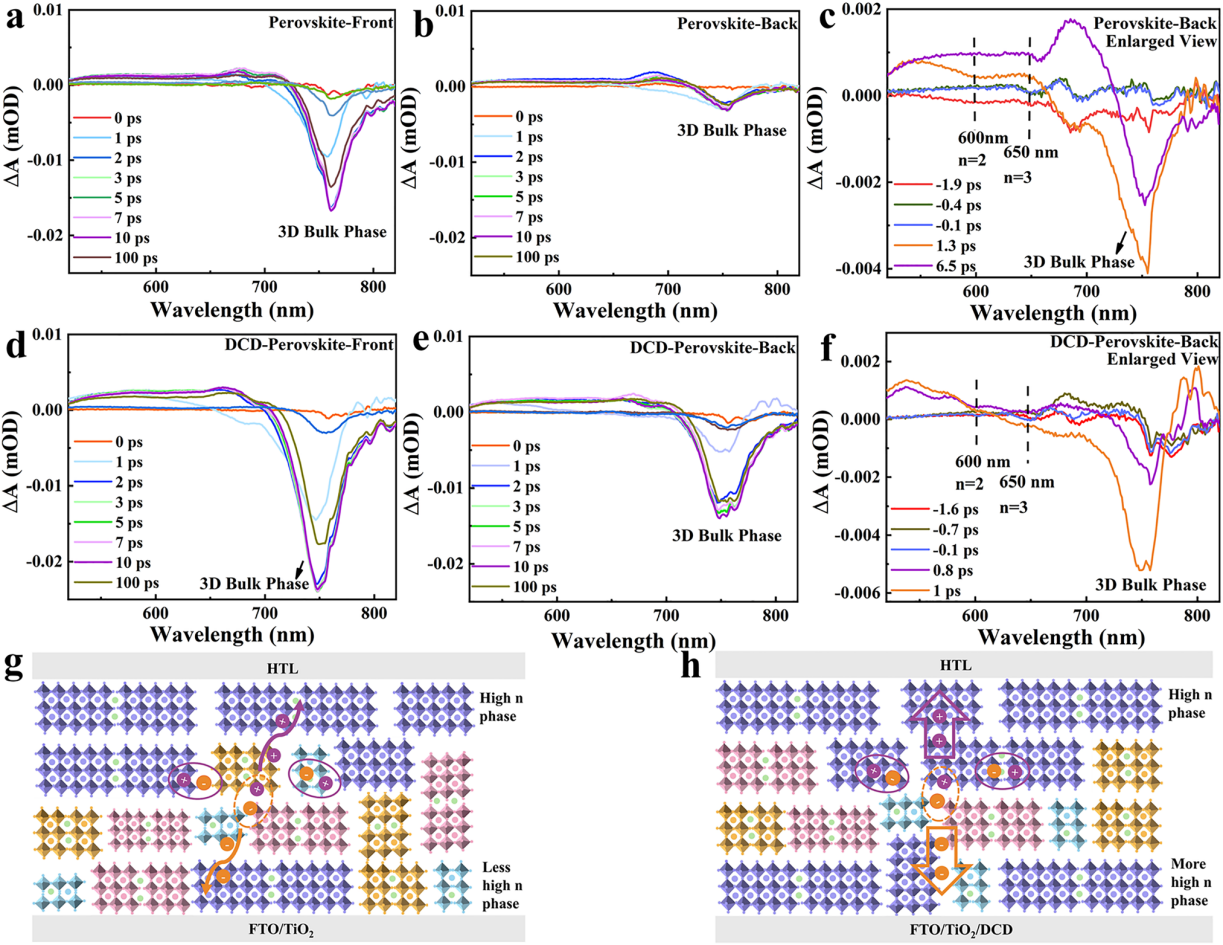
Transient absorption (TA) spectra at different delay times (0, 1, 2, 3, 5, 7, 10, and 100 ps) of the quasi-2D ACI perovskite (< *n* >  = 5) films without DCD in **a** front-side and **b, c** back-side photoexcitation. TA spectra with different delay time of the quasi-2D ACI perovskite film with DCD modified in **d** front-side and **e, f** back-side photoexcitation. **g, h** Schematic diagrams of the phase distribution of the with or without DCD film as conductive channel

Femtosecond transient absorption (TA) spectroscopy was used to investigate the phase distribution of the quasi-2D perovskite film. The perovskite film was excited from both the front and back using a 340 nm pulse laser. The TA spectra for the pure perovskite films at selected delay times are depicted in Fig. 3a, b for front-side and back-side photoexcitation, respectively. The TA spectra for the DCD-modified perovskite film are shown in Fig. 3d, e. Figure 3c, f are partial enlarged views of Fig. 3b, e, respectively. In these enlarged graphs, the ground state bleaching (GB) peaks at 600 and 650 nm correspond to *n* = 2 and *n* = 3 quantum wells (QWs), respectively, while the GB peak at 680 nm is associated with the octave peak of the excitation light. A broad GB peak centered at 750 nm indicates the presence of a 3D bulk phase. The presence of multiple GB peaks in the TA spectra suggests that the perovskite film is composed of phases with different n values. Traditionally, the quasi-2D phase distribution exhibits a trend where low-n value phases are located at the bottom of the film, while high-n value phases are preferentially found at the top, which hinders charge transport [21]. In the TA spectra of the perovskite film (Fig. 3a–c), the intensity of the 3D bulk phase peak excited from the front is significantly stronger than that excited from the back, while the GB peaks of the low-dimensional phases are barely visible on the front side. However, for the DCD-regulated perovskite film, the intensity of the 3D bulk phase peak excited from the back is slightly lower than that excited from the front but significantly higher than the 3D bulk phase peak excited from the back of the pure perovskite film. The TA results indicate that the addition of DCD improves the phase distribution of the traditional quasi-2D perovskite film, increasing the proportion of high-n-value phases at the bottom and reducing the phase distribution disparity between the top and bottom of the film.

Additionally, we measured the steady-state PL spectra of the perovskite film on a glass substrate from both front and back excitations. The intensity-normalized PL spectra for the pure perovskite and DCD-modified perovskite films are shown in Fig. S11a, b. In Fig. S11a, the peak associated with the low-dimensional phases (600 and 650 nm) are clearly visible in the curve measured from back excitation compared to the curve measured from front excitation. After adding DCD, the curves from both front and back excitations become similar, with the low n phase peaks nearly absent. The PL measurement results are consistent with the TA data, indicating that DCD regulation improves the phase distribution at the bottom of the quasi-2D film, resulting in a more uniform overall phase distribution. In the enlarged back-excited TA image, we observed that the low-dimensional peak of the control film is more prominent than that of the DCD-modified film. Notably, a small number of low-dimensional peaks in the DCD-regulated perovskite film are arranged in an orderly stepped structure, thereby reducing energy transfer losses caused by the disordered phase distribution [22]. The enhanced phase uniformity in DCD-modified perovskite films originates from *π*–*π* interactions between GAI and DCD, which suppress the formation of low-n-value phases with GA⁺ as the A-site cation while promoting the incorporation of MA⁺ at the A-site [20]. We performed in situ UV–vis absorption spectra of the wet film during the annealing process to characterize the mechanism by which DCD molecules influence the n-value distribution of perovskites (Fig. S13). The peaks of high-n phase perovskite appeared at 9.29 s in the perovskite film, while for the DCD-modified film, these peaks emerged earlier at 7.10 s. This accelerated crystallization of the high-n phase demonstrates that the *π*–*π* interactions between DCD and GA^+^ promote the formation of high-n phase structures [46]. Specifically, the addition of DCD at the buried interface increases the proportion of the 3D bulk phase at the bottom of the quasi-2D perovskite film, leading to a more uniform phase distribution across the top and bottom surfaces. This facilitates charge transport in the perovskite film and enhances the performance of PSCs (Fig. 3g, h).

### Photovoltaic Performance of Quasi-2D PSCs

**Fig. 4:**
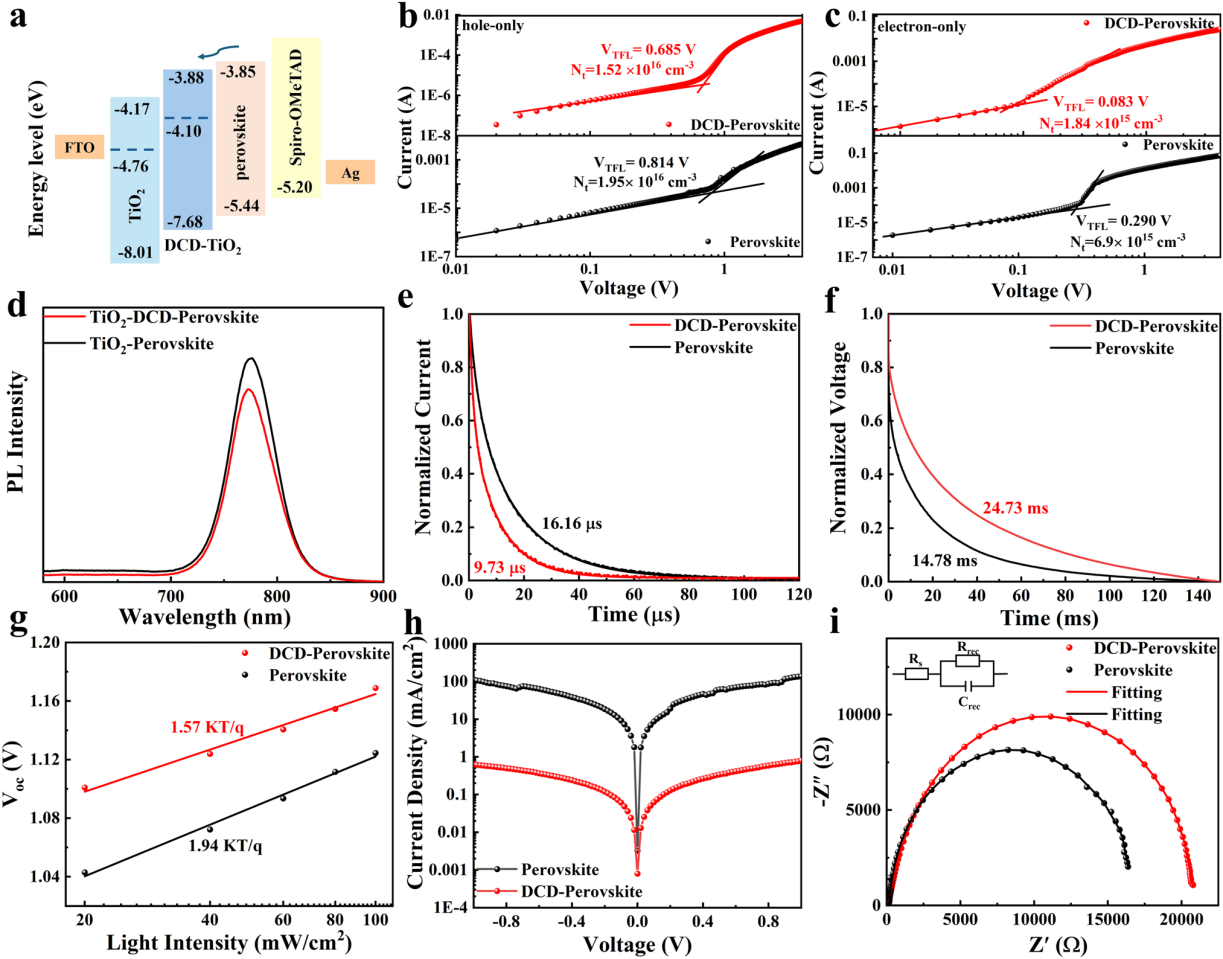
**a** Energy level diagrams of PSCs. **b, c** SCLC measurements for the DCD-modified and control devices. **d** Steady-state PL spectra of perovskite on TiO_2_ and TiO_2_-DCD. **e** Transient photocurrent (TPC) curves, **f** the transient photovoltage (TPV) curves. **g** Relationship between Voltage and Light intensity of Control and DCD modified devices. **h** The dark current–voltage curves of control and DCD modified perovskite devices. **i** Nyquist plots of PSCs

We employed space-charge-limited current (SCLC) measurement to quantitatively assess the trap state density (*N*_*t*_) of electrons and holes. The dark *J-V* curves of devices with hole-only (FTO/PEDOT:PSS/perovskite/Spiro-OMeTAD/Ag) and electron-only (FTO/TiO_2_/perovskite/PC_61_BM/Ag) structures were measured, as shown in Fig. 4b, c, respectively. By fitting the SCLC curves, we determined the defect-filled limit voltage (V_TFL_). For the DCD-modified hole-only devices, V_TFL_ was confirmed at 0.685 V, lower than the control devices with a V_TFL_ of 0.814 V. Similarly, for the DCD-modified electron-only devices, the V_TFL_ value decreased from 0.29 to 0.083 V compared to the control device. The relationship between defect density (*N*_*t*_) and V_TFL_ can be expressed by the formula [47]:

2 $$N_{t} = \frac{{\left( {2\varepsilon_{0} \varepsilon V_{TFL} } \right)}}{{\left( {eL^{2} } \right)}}$$

where ε_0_ is the vacuum permittivity, ε is the relative dielectric constant of the perovskite, e is the elementary charge, and L is the thickness of the perovskite film. After calculation, the electron *N*_*t*_ (1.84 × 10^15^ cm^−3^) and hole *N*_*t*_ (1.52 × 10^16^ cm^−3^) for the DCD-modified devices were lower than those for the control devices (6.9 × 10^15^ and 1.95 × 10^16^ cm^−3^), demonstrating that the introduction of DCD facilitates interfacial electron transport, reduces defect states, and inhibits non-radiative recombination within the PSCs, thereby enabling higher PCE. To investigate the charge transport and recombination between interfaces, we performed steady-state PL measurements on perovskite films. Figure 4d compares the PL intensity of a perovskite film on DCD-modified TiO_2_ with that on unmodified TiO_2_. The weaker PL intensity observed for the DCD-modified film suggests more rapid electron transfer between the electron transport layer (ETL) and the perovskite. This result supports the UPS findings, which indicates that DCD-modified TiO_2_ better matches the perovskite layer, enhancing charge extraction at the interfaces. Additionally, Fig. S14 displays the PL spectrum of a perovskite film on a glass substrate. Due to reduced defect-mediated recombination, the DCD-regulated perovskite film exhibits stronger PL intensity compared to the control film. Figure 4e, f presents the transient photocurrent (TPC) and transient photovoltage (TPV) characteristics of the device, respectively. The DCD-modified device exhibits a shorter charge extraction time and a longer recombination decay time, suggesting faster carrier extraction and suppressed non-radiative recombination. Furthermore, we measured the relationship between the open-circuit voltage (*V*_OC_) of the device and different light intensities (Fig. 4g). This relationship can be described by the following formula [48]:

3 $$V_{OC} = \ln \left( I \right)\frac{{nK_{B} T}}{q}$$

where K_B_ is the Boltzmann constant and q represents the elementary charge. A value of n closer to 1 indicates less trap-assisted recombination in the device. For the DCD-modified device, the n-value decreased from 1.94 to 1.57, indicating effective suppression of defect-assisted recombination. Figure 4i displays the electrochemical impedance spectroscopy (EIS) results of the device. The estimated recombination resistance (R_rec_) values of the solar cells with the pristine perovskite film and the DCD-modified perovskite film are 16,630 and 20,680 Ω, respectively [49]. The DCD-modified device exhibits higher R_rec_, confirming efficient suppression of charge recombination and contributing to an improved fill factor (FF). The dark current density–voltage curves of the PSCs are shown in Fig. 4h. The DCD-modified buried interface device exhibits smaller leakage currents, attributing to fewer defect states and suppressed carrier recombination. Furthermore, the intensity-modulated photocurrent spectroscopy (IMPS) and intense-modulated photovoltage spectroscopy analyses (IMVS) further demonstrate the efficient charge extraction and suppression of nonradiative recombination of the DCD-based devices (Fig. S15). As derived from Fig. S15 equations and listed in Table S1, the DCD-optimized devices show a remarkable carrier diffusion length (L_D_) of 732 nm, reflecting improved charge transport properties.

**Fig. 5:**
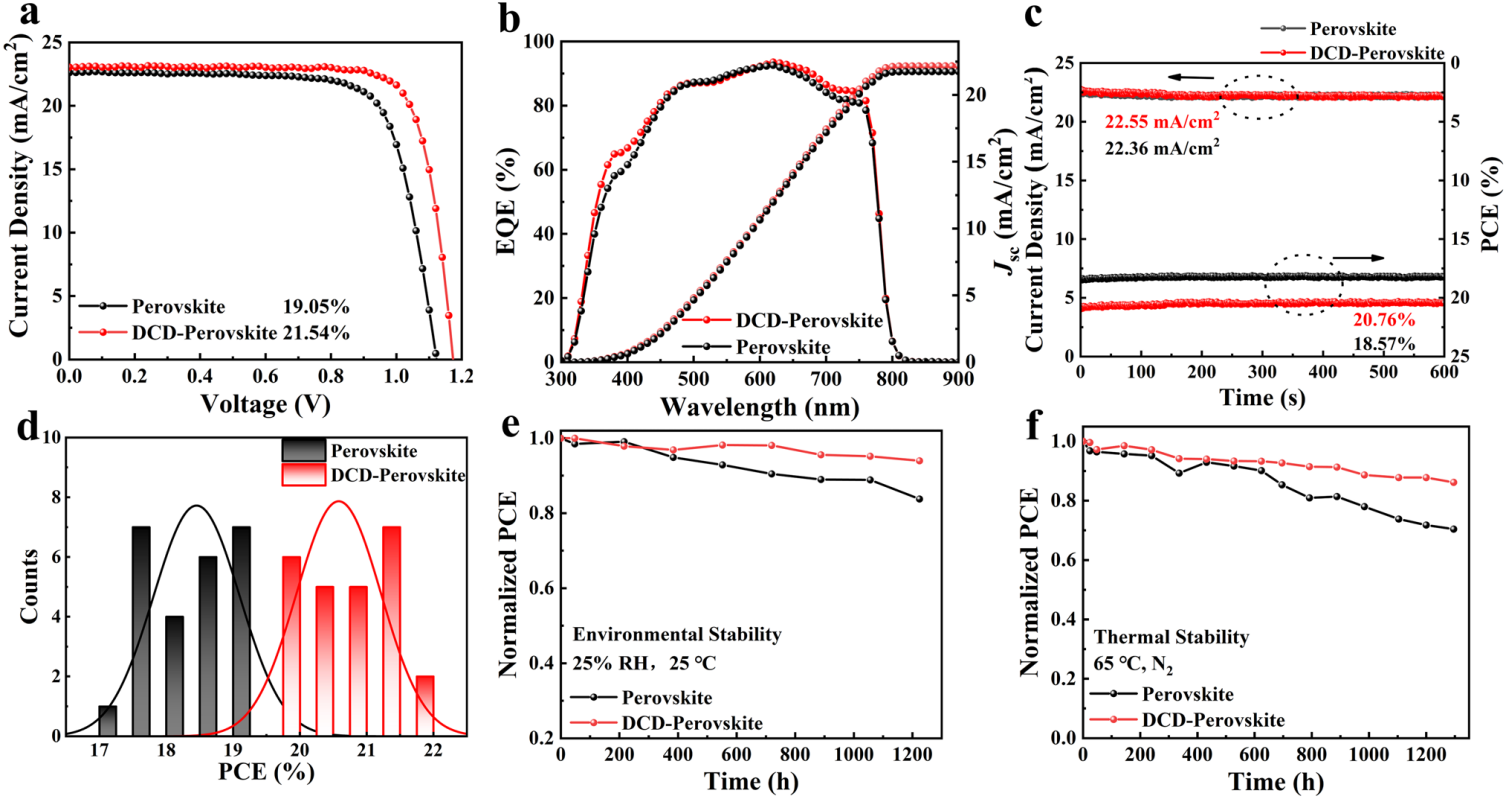
**a** *J-V* curve and efficiency data of the PSCs with and without DCD. **b** External quantum efficiency of the PSCs with and without DCD. **c** Stabilized power output of the PSCs with and without DCD treatment, measured at the maximum power point (MPP) under AM 1.5G one sun illumination. **d** Statistical distribution of PCEs obtained from 25 devices. **e** PCE evolution of unencapsulated with and without DCD PSCs stored under ambient air. **f** The stability tested at 65 °C in a nitrogen environment

The PSCs with a device structure of fluorine-doped tin oxide (FTO)/TiO_2_/with or without DCD/perovskite (GA(MA)_n_Pb_n_I_3n+1_, *n* = 5)/Spiro-OMeTAD/Ag were fabricated to evaluate their photovoltaic performance. As shown in Fig. 5a, the DCD-modified device exhibited the highest PCE of 21.54%, with an open-circuit voltage (*V*_OC_) of 1.172 V, a fill factor (FF) of 79.60%, and a short-circuit current density (*J*_SC_) of 23.08 mA cm^−2^. In contrast, the control device showed a significantly lower PCE of 19.05%, with *V*_OC_ of 1.122 V, FF of 74.93%, and *J*_SC_ of 22.64 mA cm^−2^. We also measured the external quantum efficiency (EQE) curves, as shown in Fig. 5b. The DCD-modified device demonstrated higher quantum efficiency in the wavelength range from approximately 380 and 600–750 nm compared to the control group. The integrated *J*_SC_ of the DCD-treated device was 22.17 mA cm^−2^, while the control device had 21.75 mA cm^−2^, which is closely aligned with the directly measured *J*_SC_ values. The statistical distribution of efficiencies is depicted in Fig. 5d, while the statistical distribution of *V*_OC_ and FF is shown in Fig. S16. The efficiencies, *V*_OC_, and FF of the DCD-regulated device were significantly improved due to enhanced charge transport and reduced recombination. This improvement can be attributed to decreased defects, a uniform phase distribution in the perovskite layer, and excellent interface contact between TiO_2_ and perovskite.

Additionally, we investigated the effect of the DCD-modified buried interface on device stability. Figure 5c illustrates the stabilized power output measured at the maximum power point (MPP) for 600 s. Under continuous irradiation, the *J*_SC_ and PCE of the DCD-modified device remained constant at 22.55 mA cm^−2^ and 20.76%, respectively, which are higher than those of the control device. This indicates that DCD treatment not only improves device performance but also enhances device stability. Furthermore, we measured the environmental stability (Fig. 5e) and thermal stability (Fig. 5f) of both types of devices. The DCD-treated GA(MA)_n_Pb_n_I_3n+1_ (*n* = 5)-based device maintained 94% of its initial PCE for 1200 h under ambient conditions of 25 °C and relative humidity (RH) at 25%. Under the same conditions, the pure perovskite-based device maintained only 84% of its initial PCE, which is lower than that of the DCD-modified device. We also monitored the thermal stability of PSCs with and without DCD modification at 65 °C by storing them in an N_2_-filled glove box. After 1200 h, the DCD-based PSCs and the control PSCs maintained approximately 86% and 70% of their initial PCE, respectively. The operational stability of quasi-2D PSCs was investigated under continuous full solar illumination (AM 1.5G, 100 mW cm^−2^) in a nitrogen atmosphere, as shown in Fig. S17. The control devices exhibited a decline to 71% of their initial efficiency after 200 h of continuous operation, whereas the DCD-modified PSCs retained 85% of their original efficiency after 400 h. Meanwhile, we also evaluated the perovskite film stability at 85 °C. As observed in Fig. S18, the perovskite film exhibited a distinct PbI_2_ diffraction peak at 12.6° after 200 h aging at 85 °C, indicating thermal decomposition into PbI_2_. In contrast, the DCD modified perovskite film showed negligible PbI_2_ peaks under identical conditions, demonstrating significantly enhanced stability. These results strongly suggest that DCD-modified PSCs exhibit superior heat and environmental stability, validating the significance of the perovskite buried interface passivation strategy.

## Conclusions

In this study, we have demonstrated the significant enhancement of performance in ACI-based 2D perovskite solar cells (PSCs) through the strategic incorporation of dicyandiamide (DCD) molecules. DCD plays a critical role in the effective modulation of the perovskite phase distribution, particularly by influencing the n-value composition of the 2D perovskite structure. By favoring a more uniform distribution of higher n-value phases at the bottom of the perovskite film, DCD optimizes the charge extraction process, minimizing losses from charge recombination. This modulation of the phase structure enhances the overall device efficiency by improving the energy level alignment at the buried interface, which is crucial for efficient carrier transport. Furthermore, DCD molecules induce favorable molecular conformation at the interface, facilitating better interaction between the electron transport layer (ETL) and the perovskite material. This interface engineering not only reduces interfacial defects but also enhances the interfacial charge transfer, which is critical for boosting the open-circuit voltage (*V*_OC_) and fill factor (FF). The reduction in surface roughness of the TiO_2_ layer promoted by DCD, alongside its impact on the crystallization of the perovskite film, further contributes to a smoother and more efficient interface, ensuring robust energy transport. The incorporation of DCD also leads to significant improvements in both the environmental and thermal stability of the PSCs. By stabilizing the interface structure and reducing defect states, DCD-modified devices exhibit improved operational stability under harsh conditions, making them more resilient to environmental degradation and temperature fluctuations. In summary, the DCD molecules are not only instrumental in optimizing the distribution of high n-value phases within the perovskite layer, but they also modulate the molecular conformation and interface characteristics, resulting in enhanced charge transport, minimized recombination, and superior device stability. These findings highlight the crucial role of DCD in advancing the performance and stability of 2D ACI PSCs and provide valuable insights for future interface engineering strategies aimed at developing more efficient and durable perovskite-based solar technologies.

## Supplementary Information

Below is the link to the electronic supplementary material.

Supplementary file1 (DOCX 41038 KB)
