# Supplementary material for: Dicyandiamide-Driven Tailoring of the n-Value Distribution and Interface Dynamics for High-Performance ACI 2D Perovskite Solar Cells
Source: Nanomicro Lett. 2025 Jun 23;17:305. doi: 10.1007/s40820-025-01817-x (PMC12185858; doi:10.1007/s40820-025-01817-x)
Supplement: Supplementary file 1 — Supplementary file1 (DOCX 41038 KB) [file 40820_2025_1817_MOESM1_ESM.docx]

Supporting Information for

**Dicyandiamide-Driven Tailoring of the n-Value Distribution and Interface Dynamics for High-Performance ACI 2D Perovskite Solar Cells**

Ge Chen^1, #^, Yunlong Gan^1,^ ^#^, Shiheng Wang^1^, Xueru Liu^1^, Jing Yang^1^, Sihui Peng^1^, Yingjie Zhao^1^, Pengwei Li^1,^ *, Asliddin Komilov^2^, Yanlin Song^3,^ *, Yiqiang Zhang^1,^ *

^1^ College of Chemistry, Zhengzhou University, Zhengzhou 450001, P. R. China

^2^ Karshi State Technical University, Karshi 18100; National Scientific Research Institute of Renewable Energy Sources, Tashkent 100084, Uzbekistan

^3^ Key Laboratory of Green Printing, CAS Research/ Education Center for Excellence in Molecular Sciences, Institute of Chemistry, Chinese Academy of Sciences (ICCAS), Beijing Engineering Research Center of Nanomaterials for Green Printing Technology, National Laboratory for Molecular Sciences (BNLMS), Beijing 100190, P. R. China

^#^ Ge Chen and Yunlong Gancontributed equally to this work.

*Corresponding authors. E-mail: [pwlihappy@zzu.edu.cn](mailto:pwlihappy@zzu.edu.cn) (Pengwei Li); [ylsong@iccas.ac.cn](mailto:ylsong@iccas.ac.cn) (Yanlin Song); [yqzhang@zzu.edu.cn](mailto:yqzhang@zzu.edu.cn) (Yiqiang Zhang)

**Supplementary Figures and Tables**


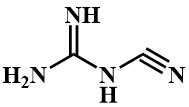


**Fig. S1** The molecular structure of Dicyanodiamide

**Fig. S2** XPS spectra of N 1S of the TiO_2_ and DCD modified TiO_2_ films

**Fig. S3** FTIR spectra of pure TiO_2_ and DCD-TiO_2_


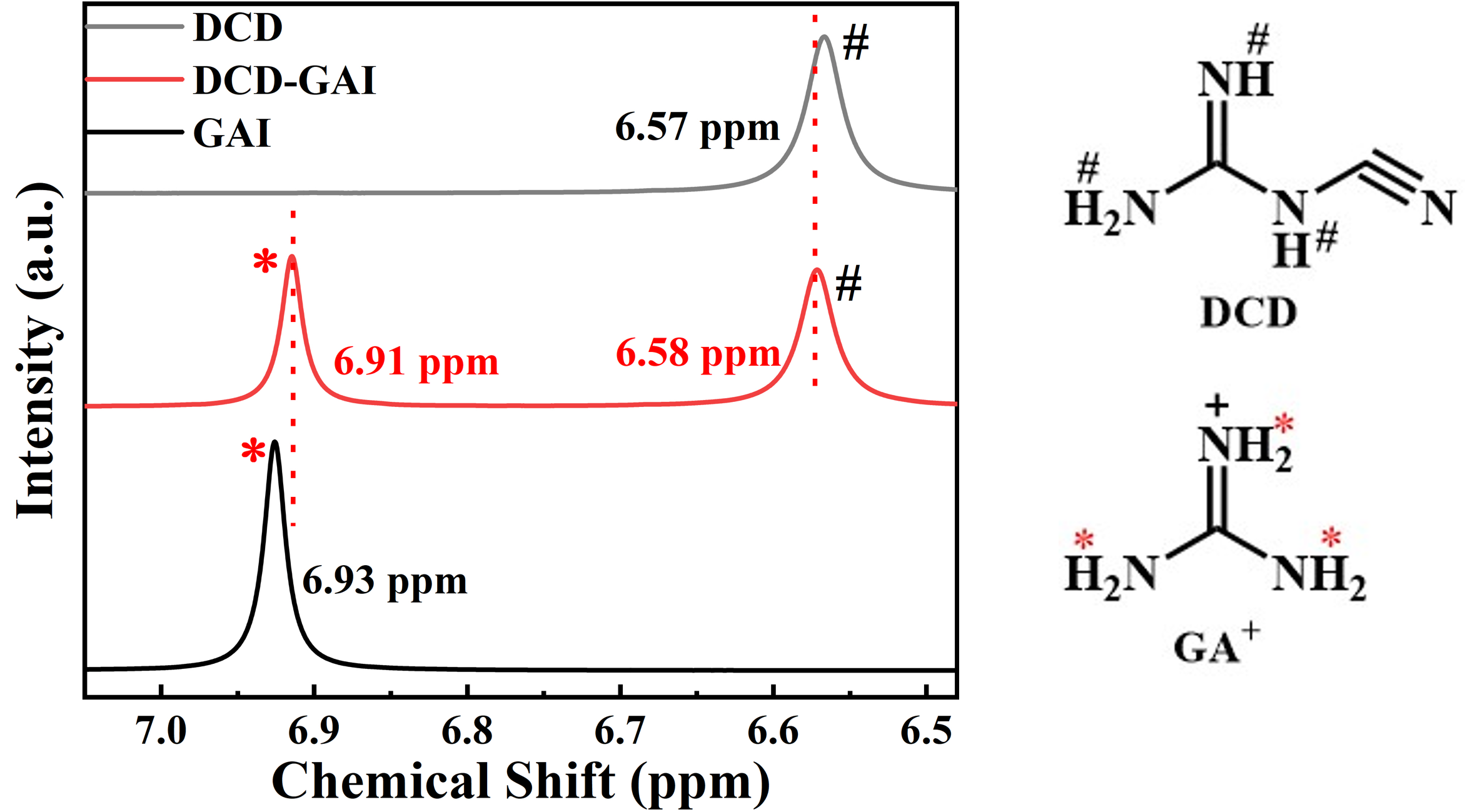


**Fig. S4** ^1^H NMR spectra of GAI, DCD and mixed powder of DCD and GAI

**Fig. S5** Formation energy of surface I vacancy without and with DCD treatment

**Fig. S6** SEM images of the bottom surface of perovskite films (**a**) without and (**b**) with DCD modification

**Fig. S7** Surface roughness of the bottom surface. (**a**) Pure perovskite film and (**b**) the DCD modified perovskite film


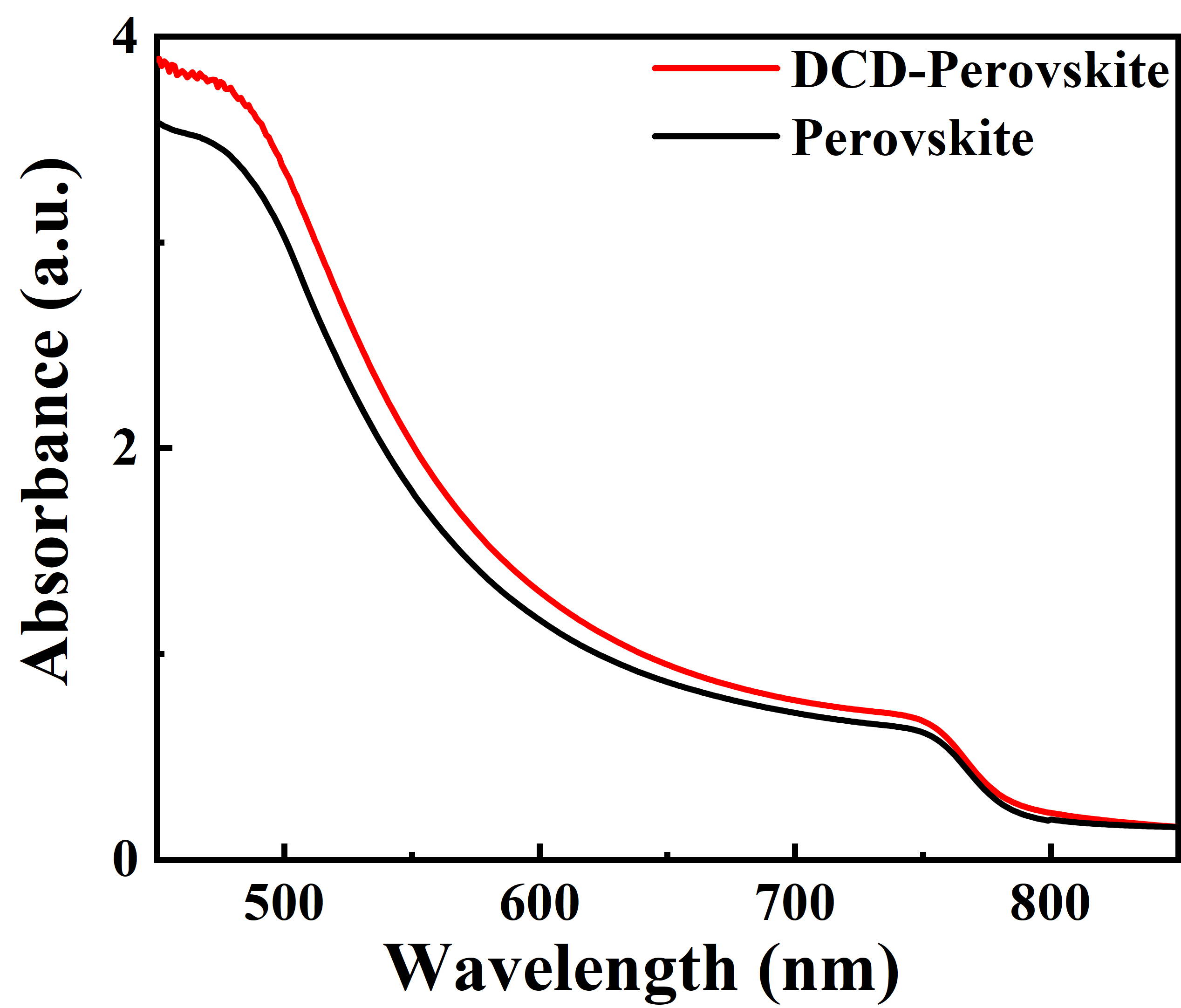


**Fig. S8** UV-vis absorption of perovskite films

**Fig. S9** AFM images of TiO_2_ and DCD modified TiO_2_ substrates, the image size is 2×2 μm

**Fig. S10** The Tauc plots of (**a**) TiO_2_ and (**b**) DCD-TiO_2_ films


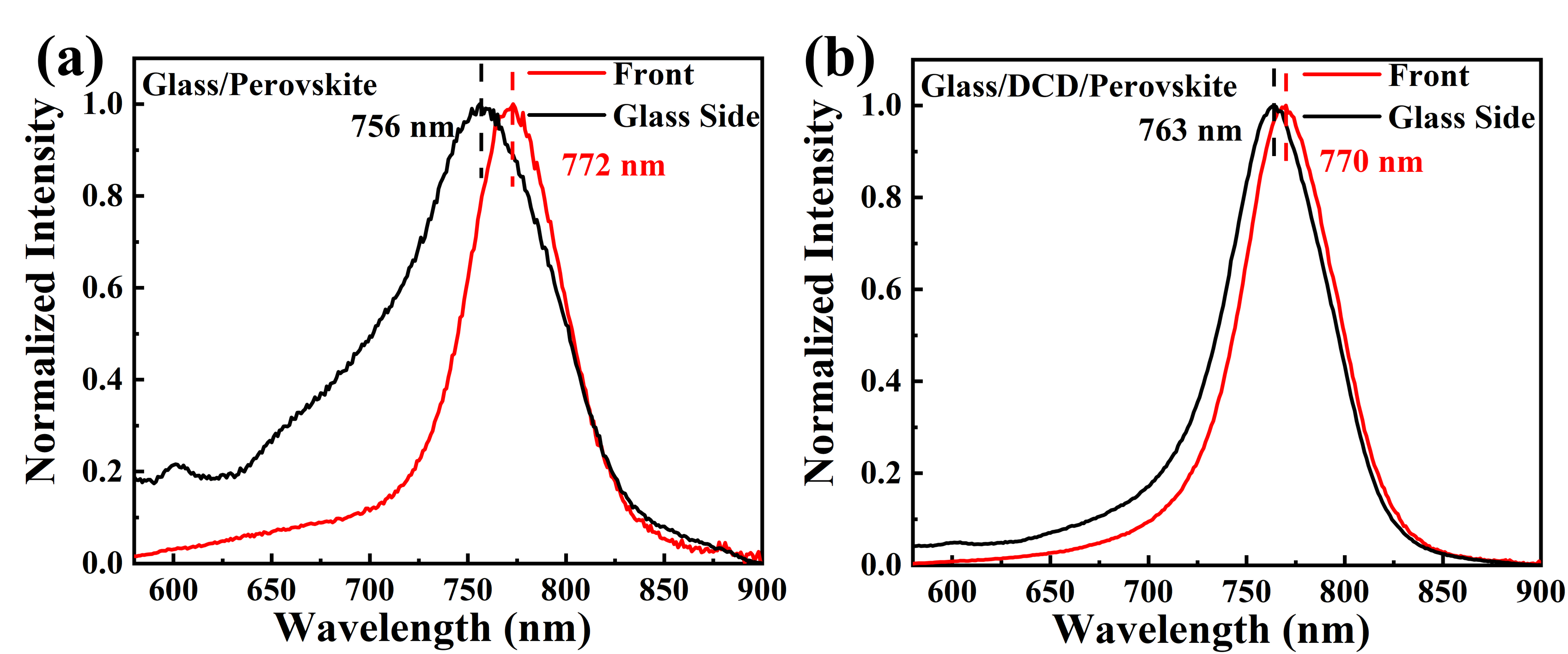


**Fig. S11** PL spectra measured from perovskite side and glass side with the control and DCD-modified perovskite films on electronic-grade glass


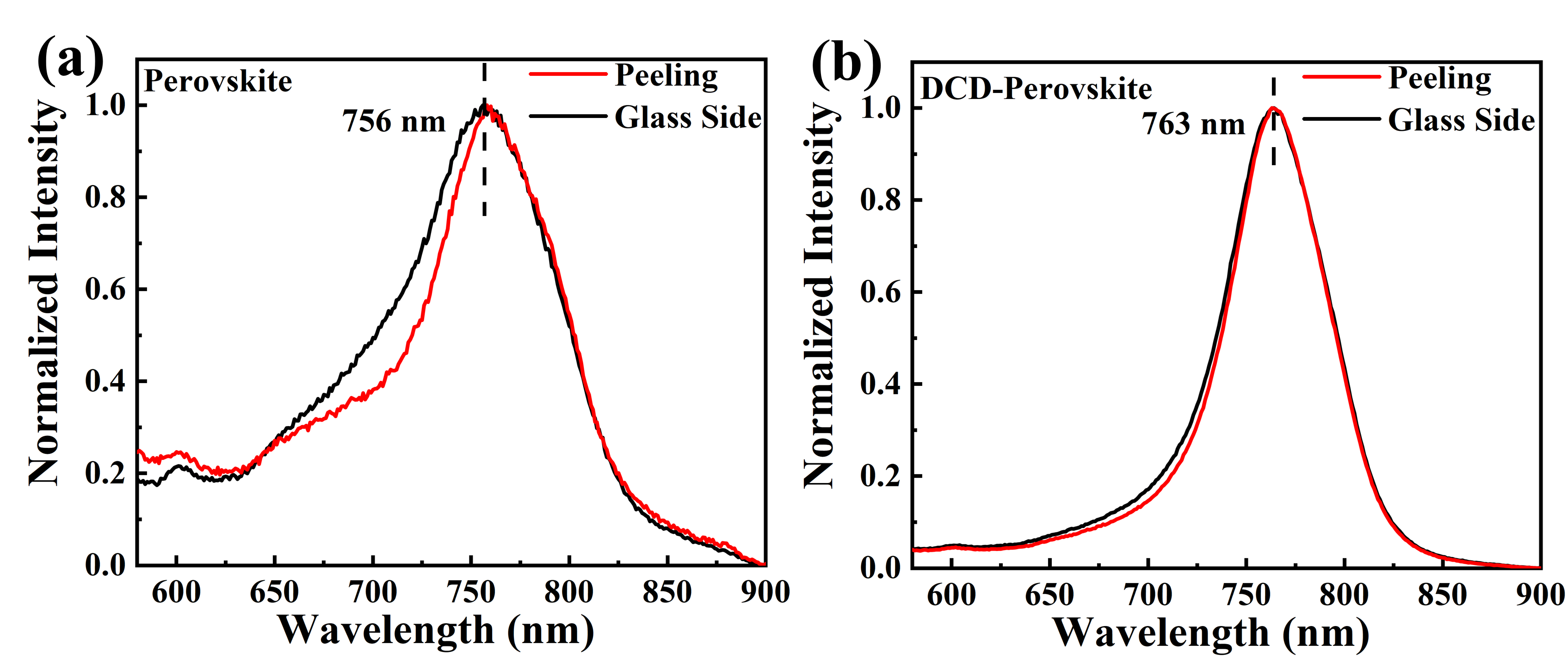


**Fig. S12** Buried-interface PL spectra of perovskite films and DCD-modified perovskite films obtained by glass side measurement and peeling method

**Note S1** The perovskite films for PL measurements were deposited on electronic-grade glass substrates. To evaluate potential interference from the glass substrate during backside measurements, we conducted comparative PL tests on both the glass-supported and peeled perovskite films. As shown in Fig. S12, the PL spectra obtained from the glass side exhibit excellent agreement with those from the peeling films, confirming negligible optical interference from the substrate.


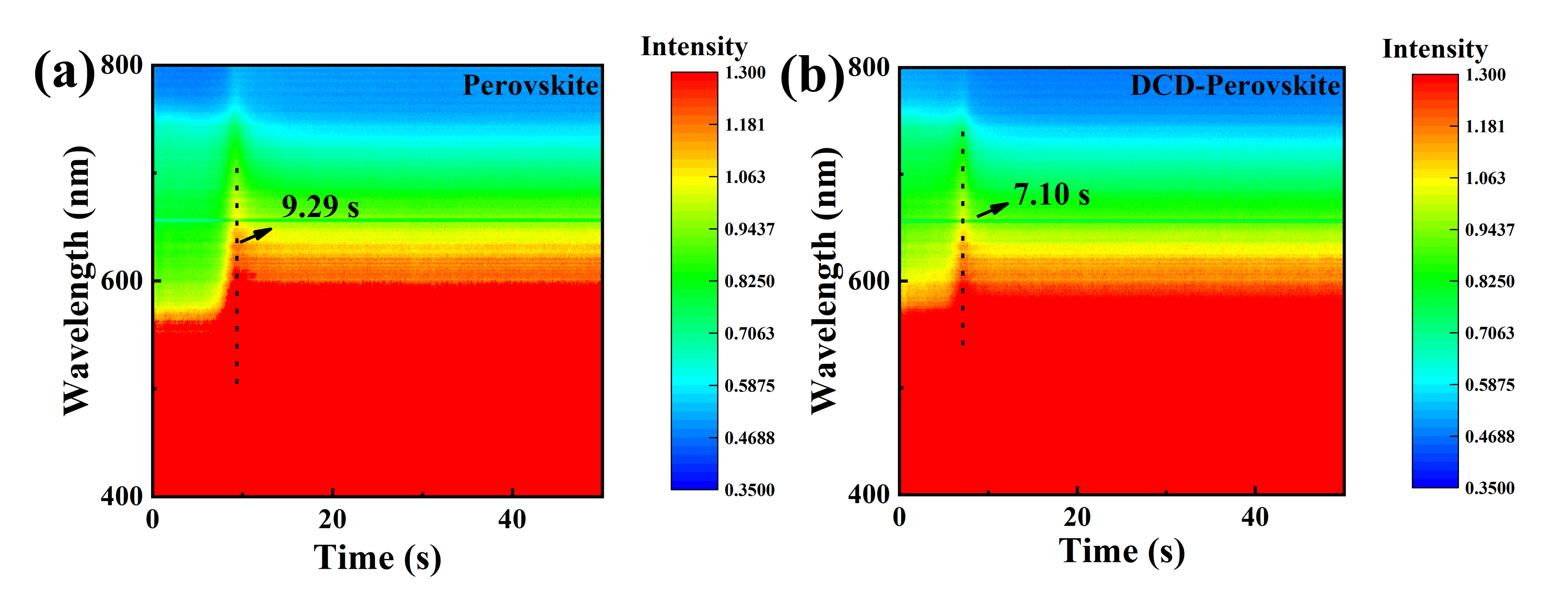


**Fig. S13** In situ UV-vis absorption spectroscopy during the annealing process of (**a**) control perovskite and (**b**) DCD-modified perovskite films


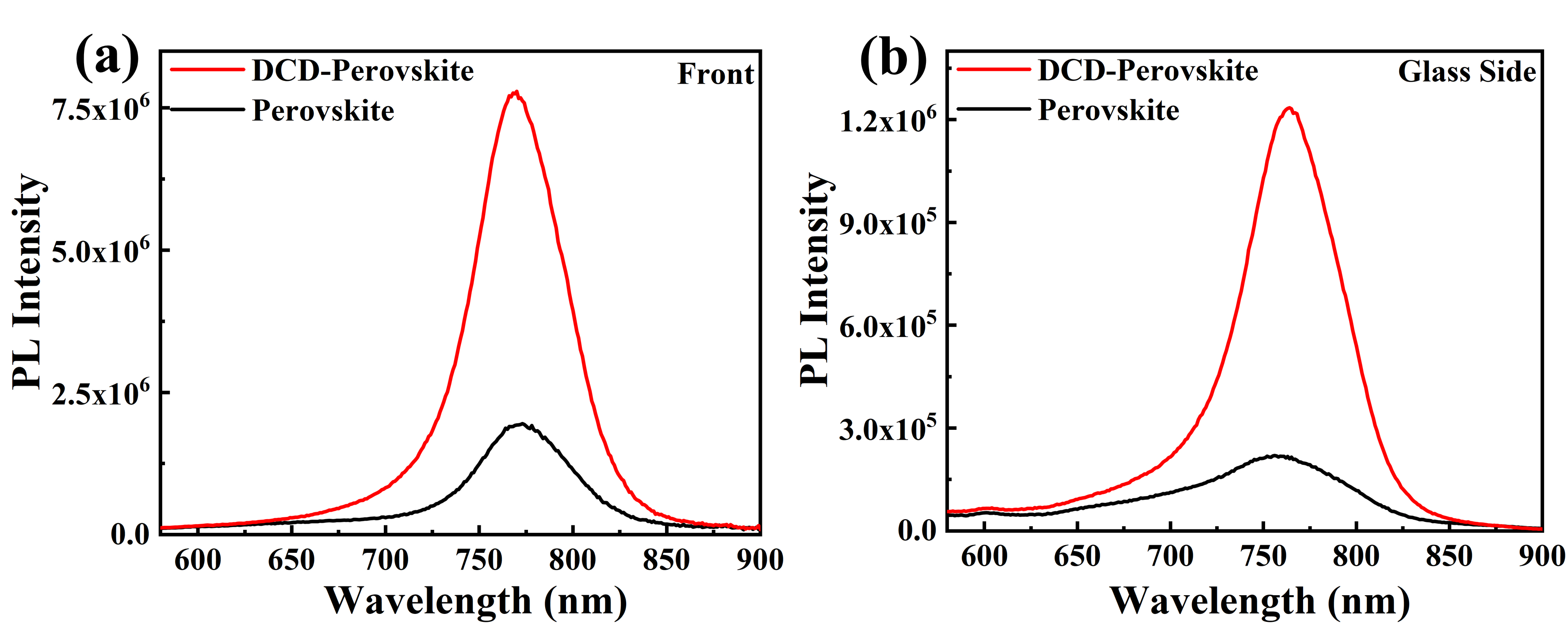


**Fig. S14** PL spectra with or without DCD passivation at the perovskite films measured from (**a**) glass side and (**b**) front


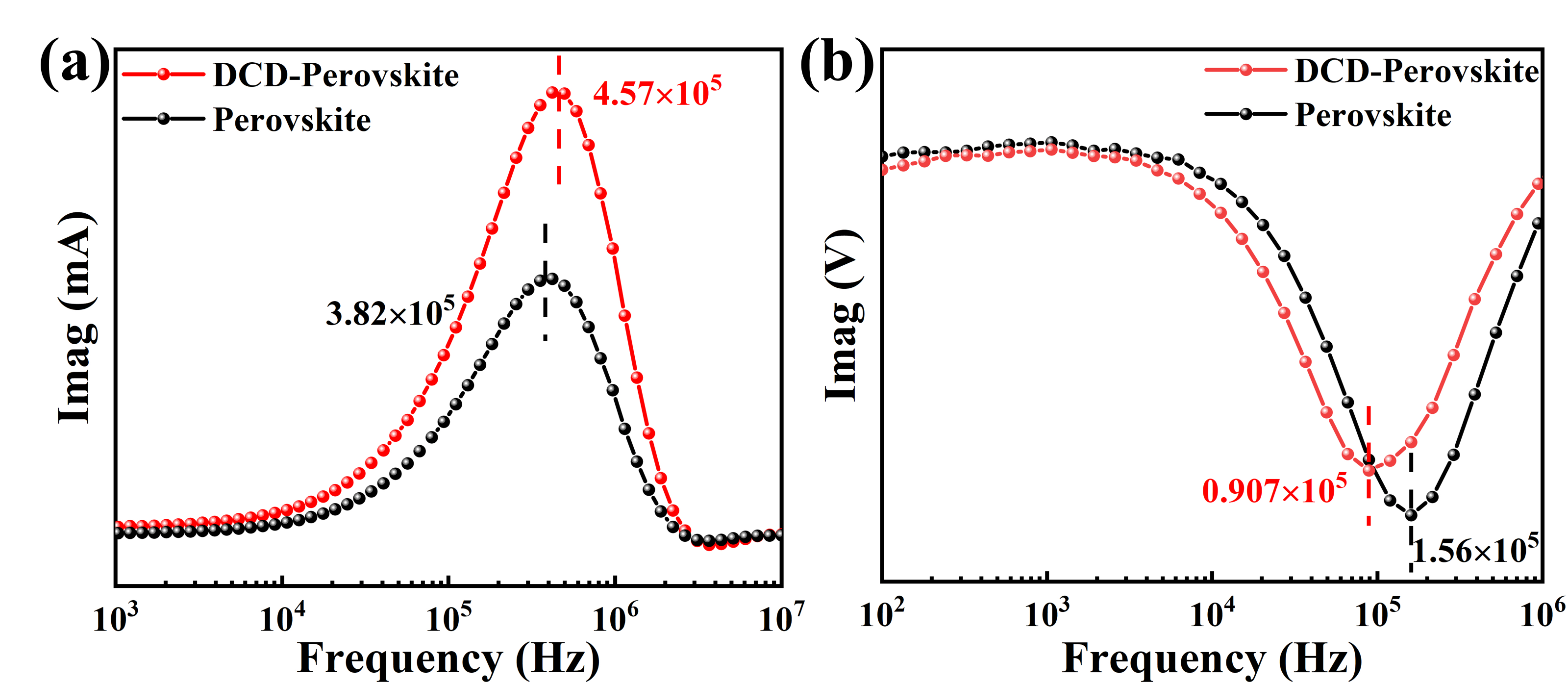


**Fig. S15 (a**) IMPS of devices based on perovskite and DCD modified perovskite, (**b**) IMVS of devices based on perovskite and DCD modified perovskite

**Calculation Method**

The carrier transport time (τ_tr_) and recombination time (τ_rec_) were derived from IMPS and IMVS spectra using Equations (S1) and (S2), respectively. Subsequently, the carrier diffusion length (L_D_) was calculated through Equations (S3) and (S4). Table 1 summarizes the effective charge diffusion lengths and relevant parameters for both perovskite and DCD-modified perovskite films.

$\tau_{tr}=\frac{2}{2\pi\times f_{IMPS}}$ (S1)

$\tau_{rec}=\frac{2}{2\pi\times f_{IMVS}}$ (S2)

$D=\frac{d^{2}}{2.35\times\tau_{tr}}$ (S3)

$L_{D}=\sqrt{D\times\tau_{rec}}$ (S4)

**Fig. S16** (**a**) *V_OC_* (**b**) FF distributions of the perovskite and the DCD modified perovskite devices


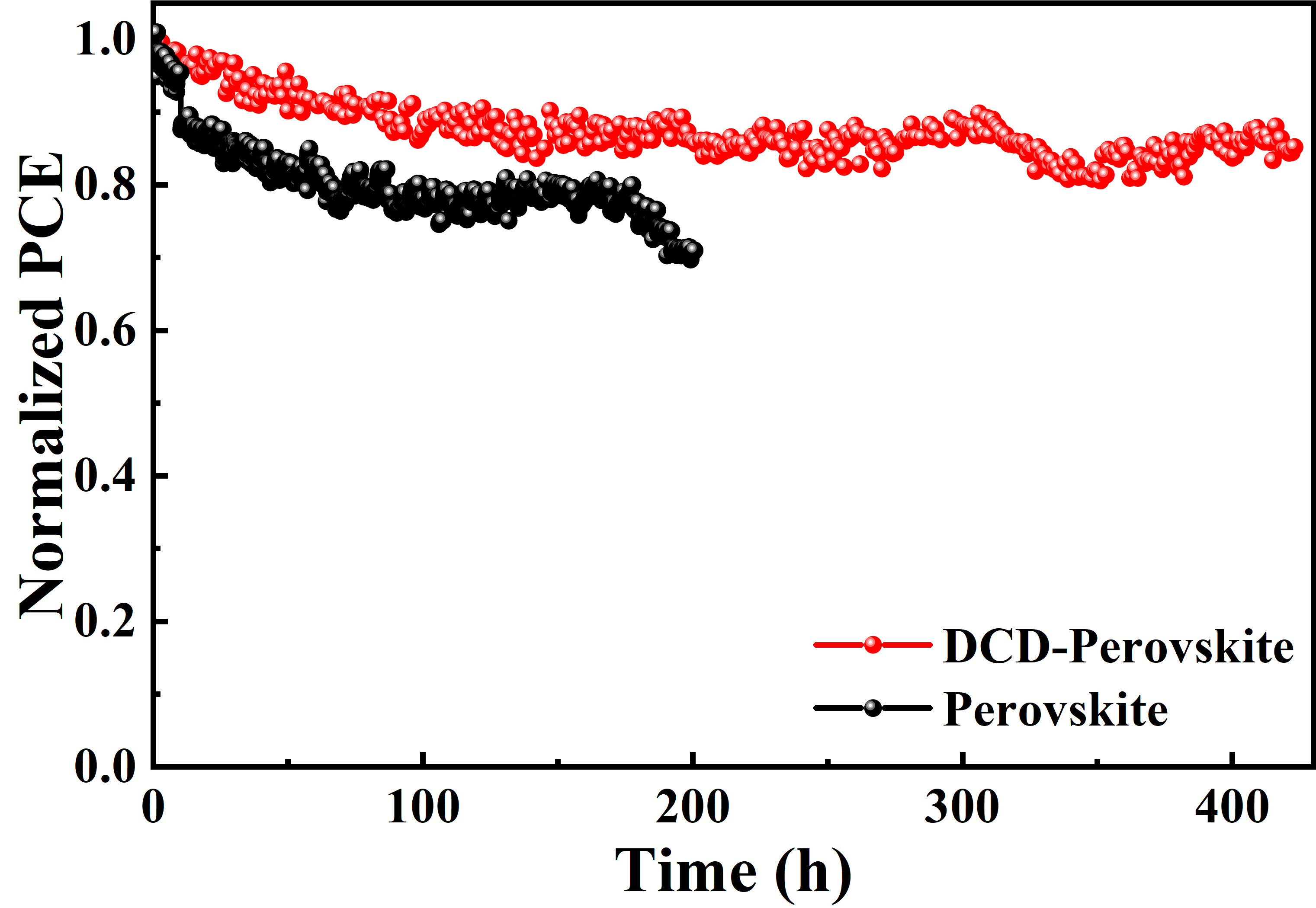


**Fig. S17** MPP tracking measured with the DCD-modified and control devices under continuous full solar illumination in a nitrogen atmosphere


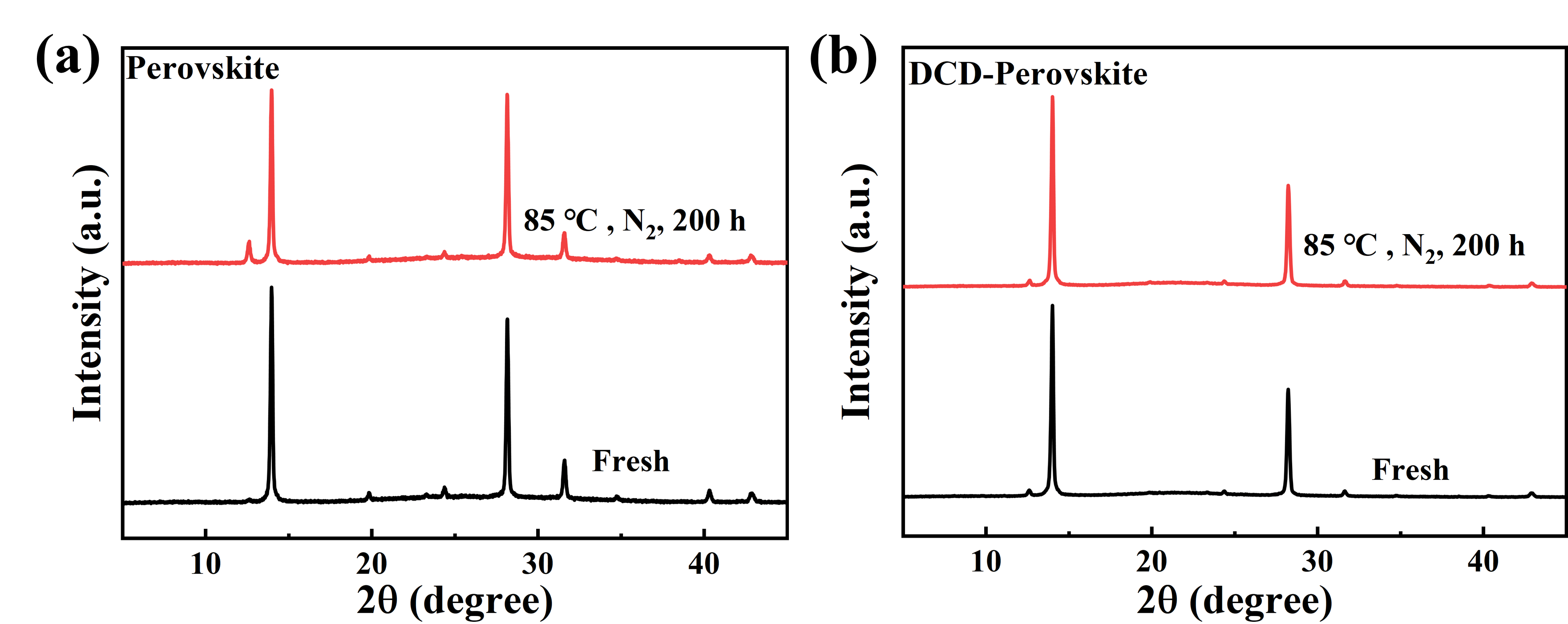


**Fig. S18** XRD patterns of (**a**) perovskite films, (**b**) DCD-modified perovskite films under high temperature conditions (85 ℃, N_2_)

**Table S1** Quantitative calculation parameters for IMPS and IMVS based on perovskite and DCD modified perovskite


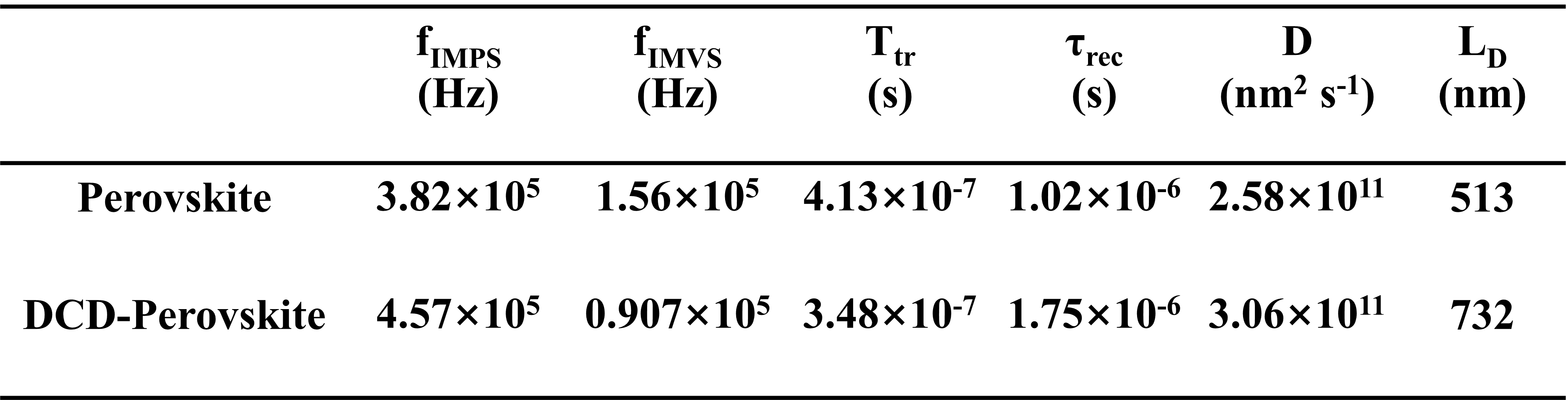


**Supplementary References**

1. G. Kresse, J. Hafner, Ab initio molecular dynamics for liquid metals. Phys. Rev. B Condens. Matter **47**(1), 558–561 (1993). <https://doi.org/10.1103/physrevb.47.558>
2. G. Kresse, J. Hafner, Ab initio molecular-dynamics simulation of the liquid-metal-amorphous-semiconductor transition in germanium. Phys. Rev. B Condens. Matter **49**(20), 14251–14269 (1994). <https://doi.org/10.1103/physrevb.49.14251>
3. J.P. Perdew, K. Burke, M. Ernzerhof, Generalized gradient approximation made simple. Phys. Rev. Lett. **77**(18), 3865–3868 (1996). <https://doi.org/10.1103/PhysRevLett.77.3865>
4. G. Kresse, D. Joubert, From ultrasoft pseudopotentials to the projector augmented-wave method. Phys. Rev. B **59**(3), 1758–1775 (1999). <https://doi.org/10.1103/physrevb.59.1758>
5. L. Shi, S. Meng, S. Jungsuttiwong, S. Namuangruk, Z.-H. Lu et al., High coverage H_2_O adsorption on CuAl_2_O_4_ surface: a DFT study. Appl. Surf. Sci. **507**, 145162 (2020). <https://doi.org/10.1016/j.apsusc.2019.145162>
